# Epigastric anterior abdominal wall hernia: An unusual cause of gastric outlet obstruction

**DOI:** 10.1016/j.radcr.2024.08.088

**Published:** 2024-09-13

**Authors:** Ramandeep Sahota, Abhishek Jayant, Rebecca Wiles, Ashok Katti

**Affiliations:** Department of Radiology, Liverpool University Hospitals NHS Foundation Trust, Mount Vernon St, Liverpool L7 8YE, England

**Keywords:** Gastric outlet obstruction, Epigastric hernia

## Abstract

A 79-year-old female presented with a 3-week history of dysphagia and vomiting, and an upper abdominal mass which had increased in size over the previous 2 weeks. CT scan showed a partial gastric outlet obstruction secondary to an epigastric hernia. This was assessed further on fluoroscopy, showing the distal stomach in the hernial sac and a delay in gastric emptying. We present the CT and fluoroscopic findings of this rare cause of gastric outlet obstruction.

## Introduction

Gastric outlet obstruction can occur secondary to any disease process that causes an impairment to gastric emptying. It can be caused by both mechanical and motility disorders, and typically presents with abdominal pain, postprandial vomiting, and weight loss. Mechanical causes in adults include peptic ulcer disease, nonsteroidal anti-inflammatory drug use, and malignancy, whilst the most common motility-related cause is gastroparesis [1].

There are previous case reports of gastric outlet obstruction secondary to a variety of hernias, including hiatus hernias, diaphragmatic hernias and inguinal hernias [[2], [3], [4]]. This case report describes a rare case of gastric outlet obstruction secondary to an epigastric anterior abdominal wall hernia, which was revealed on CT imaging; fluoroscopy confirmed the diagnosis and demonstrated the patient's functional deficit.

## Case report

A 79-year-old female presented to hospital with a 3-week history of dysphagia and vomiting. The dysphagia was associated with solid food, with episodes of vomiting occurring approximately an hour after eating. The patient described the dysphagia as feeling like food was getting stuck in her throat. Of note, she had experienced a weight loss of 2 stones over the past year.

On examination, the patient had an upper abdominal mass which had been present for a few years but had suddenly increased in size over the previous 2 weeks. The mass was nonpulsatile, and bowel sounds were heard on auscultation. The patient had not had any previous abdominal surgeries. She had underlying comorbidity of severe chronic obstructive pulmonary disease requiring home oxygen.

The main differential diagnoses for this presentation were a gastric outlet syndrome, gastric volvulus, or an oesophageal stricture. Given the significant smoking history and history of weight loss it was important to exclude malignancy. Further imaging with a CT scan was deemed appropriate to further investigate these symptoms.

**Fig. 1:**
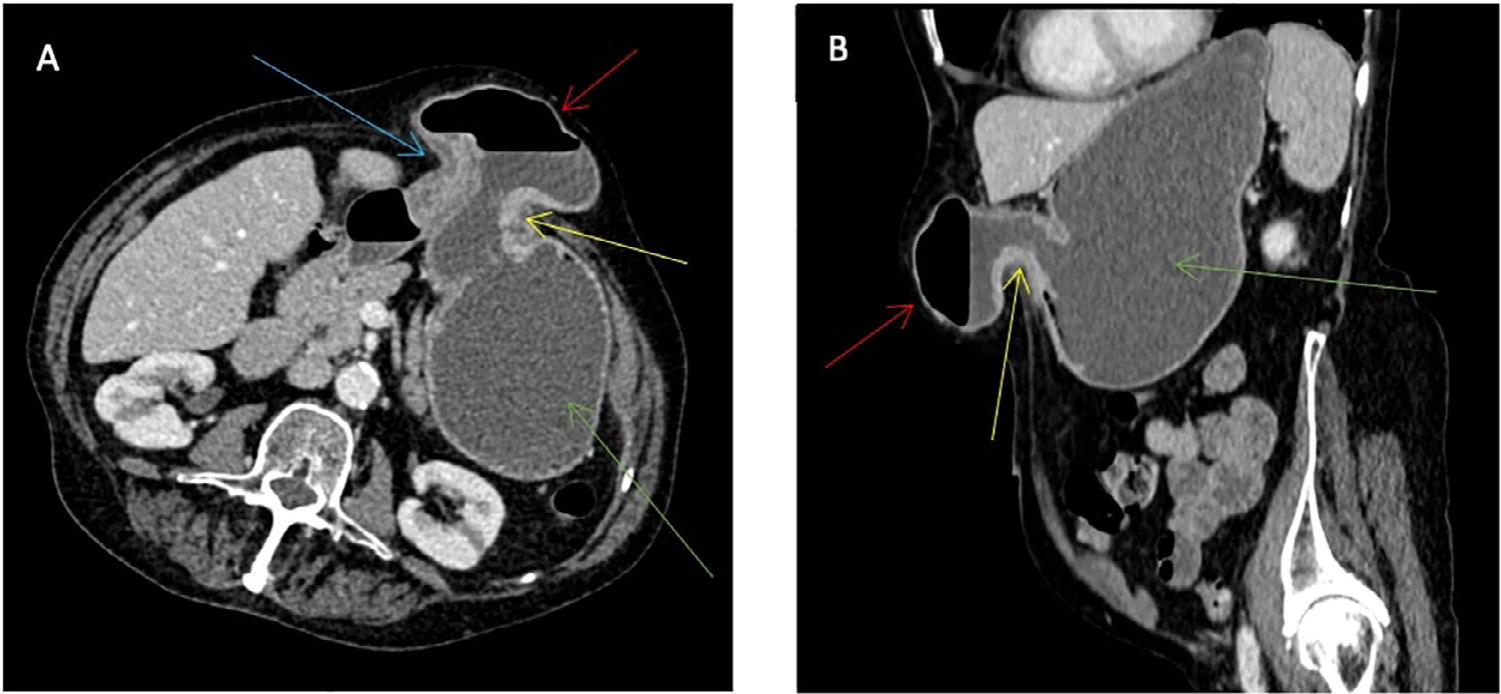
CT scan of the abdomen and pelvis with intravenous contrast in the portal venous phase. Axial (A) and coronal reformatted (B) images. These demonstrate herniation of the distal stomach through the anterior abdominal wall. The neck of the hernia is narrow and contains the distal body entering the hernial sac (yellow arrow) and the pylorus of the stomach exiting the hernial sac and re-entering the abdomen (blue arrow). There is narrowing of the lumen at both these points. The proximal stomach (green arrow) is distended, and fluid filled. The red arrow shows the hernial sac.

The initial radiological investigation was a contrast-enhanced CT scan of the chest, abdomen, and pelvis. This showed an epigastric hernia containing the gastric antrum. The neck of the hernia measured 3 cm. Proximal to the hernia, the gastric body and fundus were moderately distended. The CT appearances were suggestive of a partial gastric outlet obstruction (Fig. 1).

**Fig. 2:**
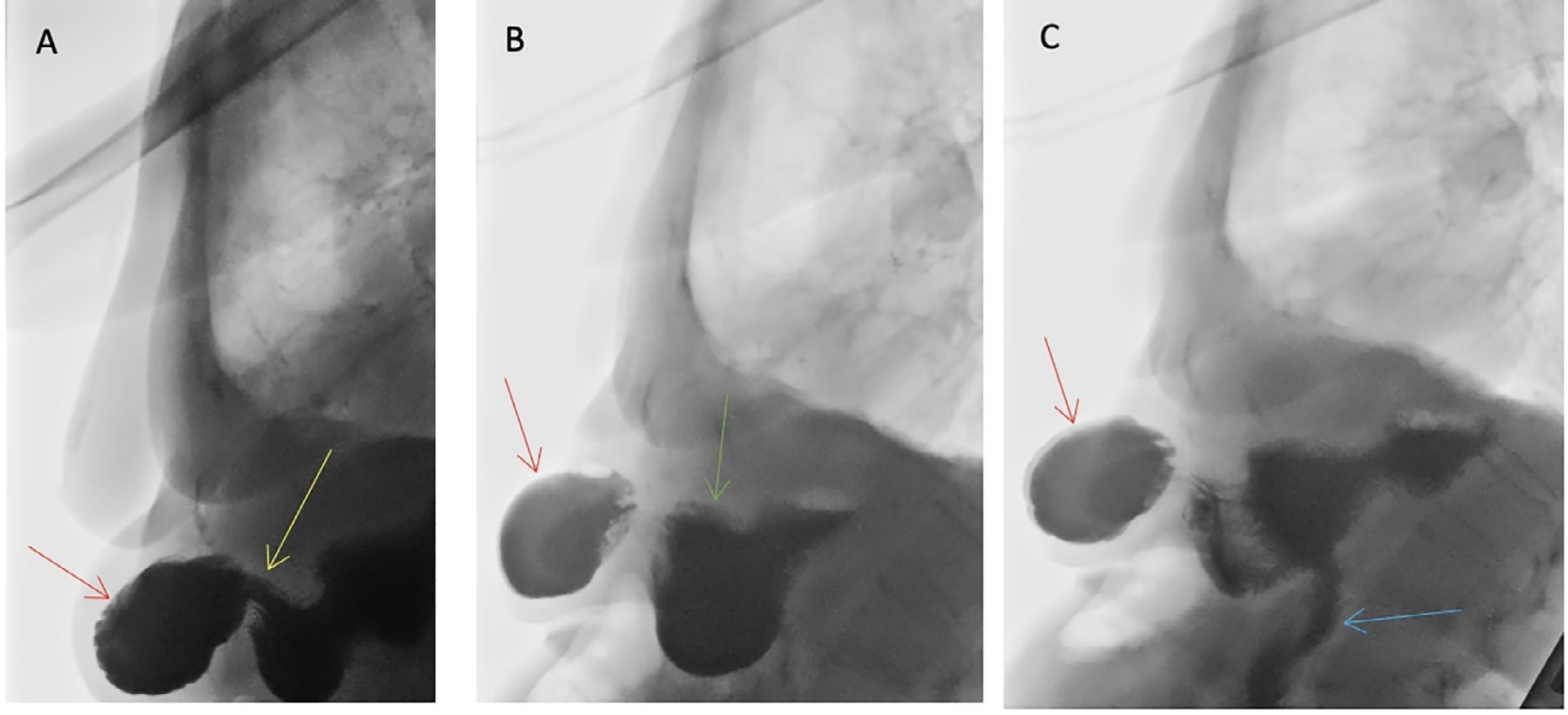
Barium swallow fluoroscopy, lateral views. (A) Significant narrowing in the distal stomach (yellow arrow) corresponding to the neck of the hernia. However, contrast is seen to reach the pylorus in the hernial sac (red arrow). (B) Hold up of contrast in the epigastric hernial sac (red arrow) and the proximal dilated stomach (green arrow). (C) Contrast entering the duodenum (blue arrow) after 9 minutes, suggesting partial obstruction.

The patient had an upper gastrointestinal tract endoscopy to insert a naso-jejunal tube. This procedure was unsuccessful due to an external compression of the gastric mucosa below the incisura angularis. Consequently, the endoscopist was unable to locate the pyloric sphincter and was therefore unable to insert the feeding tube. A contrast swallow assessment was suggested to delineate the anatomy and degree of gastric outlet obstruction (Fig. 2).

In view of her co-morbidities, the patient was not deemed a suitable candidate for surgery. Instead, the hernia was reduced conservatively with an abdominal corset brace.

Because the fluoroscopy had shown that contrast did pass through the stomach, albeit slowly, it was felt that a trial of oral feeding would be appropriate.

As an inpatient, there was regular input from dieticians to ensure the patient's nutritional requirements were being met. Nutritional bloods were monitored daily as the patient was deemed to be at high risk of refeeding syndrome. Following discharge, the patient was followed up by the community dietician to help build up from a liquid-only diet, to introducing soft solid foods. It is likely that the patient will continue to have a degree of dysphagia, though the conservative management aims to limit this.

## Discussion

Epigastric anterior abdominal wall hernias are a rare form of hernia, accounting for around 1.6%-3.6% of all abdominal hernias, and 0.5%-5% of all operated hernias [5]. They occur when tissue prolapses through a defect in the linea alba between the xiphoid process and the umbilicus [6]. They more commonly occur near the umbilicus, with epigastric hernias near the xiphoid process rare [7]. Epigastric hernias are most common in obese patients and middle-aged men [6]. Other risk factors include coughing, chronic constipation, and structural deformities [6]. The hernias usually contain preperitoneal fat, with the prolapse of intra-abdominal viscera into the hernial sac a rare occurrence [8].

Whilst many patients remain asymptomatic, symptomatic presentations of an epigastric hernia include abdominal pain, increased food intake, and dyspepsia [8,9]. These symptoms are nonspecific, making diagnosis challenging. This is exacerbated if physical examination is not possible, or if the patient does not have an obvious abdominal wall defect. A prompt diagnosis is imperative as complications of epigastric hernias include gastric ischemia, which requires immediate surgery. Though rare, mortality as a direct result of gastric ischemia is approximately 24% within 6 months [10]. Other emergency complications of an epigastric hernia include incarceration and gangrenous contents, both requiring immediate surgery [6].

The diagnosis of epigastric hernia is often made on clinical history; however, imaging can be a useful adjunct in less certain cases. An urgent CT is usually the imaging modality of choice, though this case report shows the value of a fluoroscopic study in assessing the functional deficit secondary to a gastric outlet obstruction. CT scans are useful to confirm the diagnosis and the contents of the hernia, assess for complications, and assist in any surgical planning.

The management of epigastric hernias are directed by patient symptoms, and can be either surgical, or as in the example of this case report, conservative. Surgical management in an uncomplicated epigastric hernia involves reduction of the hernial contents with repair of the rectus sheath, augmented with mesh placement [11]. A complicated epigastric hernia often requires emergency surgery, and may also involve bowel resection and anastomosis [8].

There have been only a few previous case reports describing cases of epigastric hernias with stomach as content [9,12]. The unique aspect of this case report is that it images an epigastric hernia with gastro-oesophageal obstruction containing stomach with both CT and fluoroscopy.

## Conclusion

Whilst an epigastric hernia containing stomach content is rare, the diagnosis can be considered in patients presenting with dysphagia, vomiting, and a new epigastric abdominal wall mass. Prompt diagnosis is critical, and imaging with an urgent CT can help to determine the evolution of the disease process and the potential risk of complications. Dynamic studies such as fluoroscopy are a useful adjunct to a CT scan to determine the functional deficit to the patient because of a gastric outlet obstruction and help to guide their management, particularly in patients in whom nonsurgical intervention is being considered.

## Patient consent

Written informed consent was obtained from the patient for publication of this case review, including accompanying images.
